# Editorial: Population Pharmacogenomics (PGx): From Variant Identification to Clinical Implementation

**DOI:** 10.3389/fgene.2021.736626

**Published:** 2021-08-05

**Authors:** Masahiro Hiratsuka, Yitian Zhou, Volker M. Lauschke

**Affiliations:** ^1^Graduate School of Pharmaceutical Sciences, Tohoku University, Sendai, Japan; ^2^Department of Physiology and Pharmacology, Karolinska Institutet, Stockholm, Sweden; ^3^Dr. Margarete Fischer-Bosch Institute of Clinical Pharmacology, Stuttgart, Germany

**Keywords:** population genetics, pharmacogenomics (PGx), precision medicine, CYP, N-acetyltransferase (NAT)

It is by now well-established that genetic differences underlie the inter-individual variability in pharmacokinetics, response, and toxicity of many drugs. Gene families of particular interest in this context include those encoding cytochrome P450 enzymes (CYPs), other drug metabolizing enzymes, such as N-acetyltransferases (NATs), DPD, and TPMT, as well as drug transporters of the ATP-binding cassette (ABC) and solute carrier (SLC) superfamilies (Lauschke et al., 2017, 2019; Roden et al., 2019). In total, associations between germline polymorphisms and drug-related phenotypes are established for more than 200 drugs and have been included into the respective labels. Well-established and mechanistically understood examples include links between *DPYD* and *TPMT* genotype with fluoropyrimidine and thiopurine toxicity, respectively, associations of CYP2D6 and CYP2C19 metabolizer status with the response to various anti-depressants and anti-psychotics, as well as correlations between variations in human leukocyte antigen (*HLA*) genes encoding the major histocompatibility complex and severe hypersensitivity reactions to abacavir, carbamazepine, and allopurinol.

To render the implementation of the testing of such pharmacogenomic biomarkers into routine clinical care a cost-effective allocation of health care resources, it is important to know, besides other parameters, the population-specific frequency of the polymorphisms in question. For instance, previous research showed that preemptive testing of HLA-B^*^15:02 of 50–150 patients was sufficient to prevent one adverse drug reaction (ADR) due to carbamazepine in China and South-East Asia, whereas >10,000 individuals would need to be tested in Japan, or throughout Africa and Europe (Zhou et al., 2021). As a consequence, preemptive HLA-B^*^15:02 genotyping is only cost-effective for individuals of South-East Asian ancestry. Similarly, we and others have shown striking ethnogeographic differences for multiple polymorphisms in *CYPs*, drug transporters, *DPYD*, and *TPMT* (Gordon et al., 2014; Fujikura et al., 2015; Mizzi et al., 2016; Zhou et al., 2017, 2020; Schaller and Lauschke, 2019; Petrovic et al., 2020; Xiao et al., 2020; Runcharoen et al., 2021). While these studies provided an important first step, they only considered seven global populations. Thus, further efforts which map the relevant pharmacogenomic variability with higher population resolution can be expected to facilitate the guidance of refined strategies to guide genotype-informed care.

In this Research Topic, Fukunaga et al. mapped the population-specific frequencies of *NAT2* alleles and experimentally characterize their functional consequences. Specifically, the authors analyzed the frequencies of NAT2^*^4, ^*^5, ^*^6, and ^*^7 based on genetic data from 990 Japanese individuals and compared results to available frequency information from the 1000 Genomes Project populations. Furthermore, they experimentally determined *Km, Vmax*, and *CLint* of these alleles using eight different model substrates. Based on these data, the authors concluded that frequencies of slow or ultra-slow acetylators, i.e., those carrying one or more ^*^5, ^*^6, or ^*^7 alleles, was between 30 and 55% in Europeans, Africans and South Asians, whereas the prevalence of slow acetylator phenotypes in Japanese and other East Asians was substantially lower (4–11%).

In an additional study, Zhang et al. analyzed the patient benefits of the implementation of *CYP2C19* genotyping for the guidance of antiplatelet therapy in China. In this observational study of patients undergoing percutaneous coronary intervention, clopidogrel, or ticagrelor was recommended to be prescribed depending on the absence or presence of *CYP2C19* loss-of-function (LOF) alleles (^*^2 or ^*^3), respectively. While cardiologists mostly adhered to the pharmacogenetic recommendations, those patients with *CYP2C19* LOF alleles that were prescribed clopidogrel in opposition to the pharmacogenetic recommendation had significantly higher rates of major cardiac or cerebrovascular adverse events (7.8 vs. 4.0%; *p* = 0.029). No significant differences in major bleeding events were observed between genotype and treatment groups. These results are particularly important as the currently available evidence regarding the benefits of pharmacogenomics-guided treatment for cardiovascular diseases is limited with mixed results (Zhu et al., 2020).

Lastly, an interesting study by the Human Heredity and Health in Africa (H3Africa) Consortium provides an overview of the pharmacogenomic variation in Sub-Saharan Africa based on 458 high-coverage whole genome sequences. The authors find drastic differences in population frequencies between the different ethnogeographic groups and identify 930 single nucleotide variants (SNVs) with putative functional consequences, most of which were restricted to specific populations. Together with other studies (Radouani et al., 2020; Pernaute-Lau et al., 2021), this resource increases the available information about the pharmacogenetic diversity in Africa considerably and incentivizes functional testing of the identified variants in question.

In summary, we are confident that the papers included in this Research Topic increase our understanding of pharmacogenomic population diversity and provide useful information for the optimization and facilitation of population-specific precision public health efforts in previously understudied populations.

## Author Contributions

All authors contributed to the writing and approved the final version of the manuscript.

## Conflict of Interest

YZ and VL are co-founders and shareholders of PersoMedix AB. In addition, VL is CEO and shareholder of HepaPredict AB and discloses consultancy work for Enginzyme AB. The remaining author declares that the research was conducted in the absence of any commercial or financial relationships that could be construed as a potential conflict of interest.

## Publisher's Note

All claims expressed in this article are solely those of the authors and do not necessarily represent those of their affiliated organizations, or those of the publisher, the editors and the reviewers. Any product that may be evaluated in this article, or claim that may be made by its manufacturer, is not guaranteed or endorsed by the publisher.
